# Specific inhibition of one DNMT1-including complex influences tumor initiation and progression

**DOI:** 10.1186/1868-7083-5-9

**Published:** 2013-06-28

**Authors:** Mathilde Cheray, Romain Pacaud, Arulraj Nadaradjane, François M Vallette, Pierre-François Cartron

**Affiliations:** 1 Centre de Recherche en Cancérologie Nantes-Angers, INSERM, U892, Equipe Apoptose et progression tumorale, Equipe labellisée Ligue Nationale Contre le Cancer, 8 quai moncousu, BP7021, Nantes, 44007, France; 2 Département de Recherche en Cancérologie, Université de Nantes, Faculté de Médecine, IFR26, Nantes, F-4400, France; 3 LaBCT, Institut de Cancérologie de l'Ouest, Boulevard J Monod, Nantes, Saint Herblain Cedex, 44805, France; 4 Institut de Recherche Thérapeutique INSERM U892 – CRCNA, Equipe 9 –Apoptose et Progression tumorale, 8 Quai Moncousu, BP 70721, Nantes, Cedex 1, 44007, France

**Keywords:** DNMT1, Epigenetic, DNA methylation, Glioma, GBM, Cell death

## Abstract

**Background:**

Reactivation of silenced tumor suppressor genes by DNMT inhibitors has provided an alternative approach to cancer therapy. However, DNMT inhibitors have also been shown to induce or enhance tumorigenesis via DNA hypomethylation-induced oncogene activation and chromosomal instability. To develop more specific DNMT inhibitors for efficient cancer therapy, we compared the effects of peptides designed to specifically disrupt the interaction of DNMT1 with different proteins.

**Findings:**

Our data indicated that the use of an unspecific DNMT inhibitor (5aza-2deoxycytidine), a DNMT1 inhibitor (procainamide) or peptides disrupting the DNMT1/PCNA, DNMT1/EZH2, DNMT1/HDAC1, DNMT1/DNMT3b and DNMT1/HP1 interactions promoted or enhanced in vivo tumorigenesis in a mouse glioma model. In contrast, a peptide disrupting the DNMT1/DMAP1 interaction, which per se did not affect tumor growth, sensitized cancer cells to chemotherapy/irradiation-induced cell death. Finally, our data indicated that the peptide disrupting the DNMT1/DMAP1 interaction increased the efficiency of temozolomide treatment.

**Conclusion:**

Our data suggest that the DNMT1/DMAP1 interaction could be an effective anti-cancer target and opens a new avenue for the development of new strategies to design DNMT inhibitors.

## Findings

Numerous reports have identified the occurrence of global DNA hypomethylation as an oncogenic event in human tumorigenesis. Indeed, DNMT1 silencing and the disruption of the DNMT1/PCNA/UHRF1 complexes were described as two events that promote the initiation of tumorigenesis (malignant transformation from a non-tumor cell to tumor cell) via the induction of global DNA hypomethylation [1-4]. However, besides PCNA and UHRF1 proteins, DNMT1 has multiple partners of interaction, the loss of which could potentially promote the initiation of tumorigenesis or progression via the generation of the global DNA hypomethylation phenotype.

### Disruption of certain DNMT1/protein-x interactions

**Figure 1:**
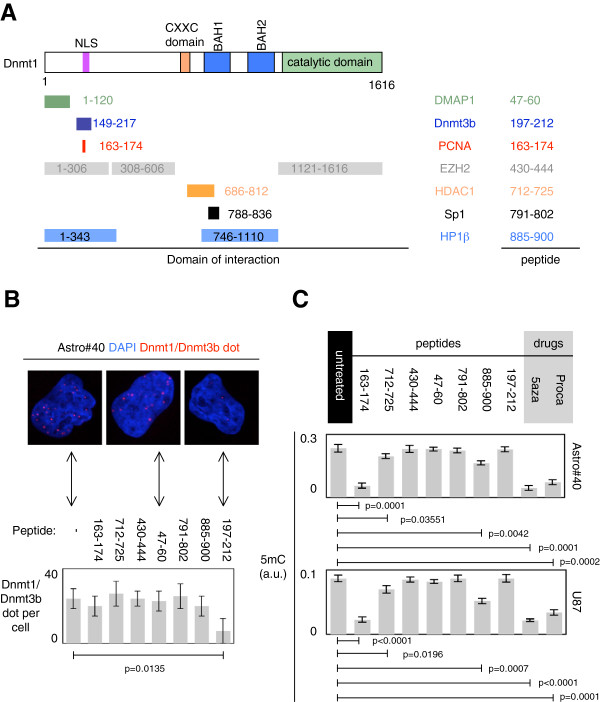
**Specific disruption of DNMT1/protein-x interactions.** (**A**) Representation of DNMT1 with its functional domain (nuclear localization signal: NLS, BAH1 and two domains, domain CXXC and its catalytic domain) and with amino acid domains of interaction with DMAP1, HDAC1, Sp1, HP1β, EZH2, DNMT3b and PCNA according to the UniProt website and the literature. Amino acid sequences of peptides cloned in plasmid and able to disrupt specific DNMT1/protein-x interactions are indicated in the “*peptide*” column; “*untreated*” indicates that cells were not treated with a peptide encoding by a plasmid, i.e., that cells were only treated with a plasmid encoding for the NLS of interest. (**B**) Only the expression of the 197–212 peptide promotes a significant decrease of the DNMT1/DNMT3b interaction in Astro#40 cells. Pictures (*blue:* DAPI staining; *red:* DNMT1/DNMT3b interaction or close proximity) illustrate the specific disruption of the DNMT1/DNMT3b interaction or close proximity induced by the expression of the 197–212 peptide, while the expression of the 47–60 peptide did not affect the DNMT1/DNMT3b interaction or close proximity. Pictures are representatives of data obtained from 100 cells in three independent experiments. The graph illustrates the average ± SD of these data. (**C**) Measure of the 5-methylcytosine (5 mC) level in cells (Astro#40 or U87) transfected by plasmid encoding for the indicated peptides or pre-treated with procainamide (0.5 mmol/l each 5 days for 4 weeks) or 5aza-2deoxycytidine (1μM each 5 days for 4 weeks).

Among the DNMT partners, we focused our analysis on the impact of tumorigenesis on the disruption of the DNMT1/PCNA, DNMT1/HDAC1, DNMT1/HP1β, DNMT1/EZH2, DNMT1/Sp1, DNMT1/DMAP1 and DNMT1/DNMT3b complexes (Figure 1A) [5-14].

To investigate the impact of the disruption of these interactions on tumorigenesis, we transfected an astrocyte cell line (Astro#40) and a glioma cell line (U87) with plasmid constructions encoding for peptides mimicking certain amino-acid regions implicated in the interactions existing between DNMT1 and DMAP1, DNMT3b, PCNA, EZH2, HDAC1, Sp1 and HP1β (Figure 1A and Additional file 1: Figure S1).

After 4 weeks of transfection/selection/amplification of cells, proximity ligation in situ assays (P-LISA) revealed that the 47–60, 197–212, 163–174, 430–444, 712–725, 791–802 and 885–900 peptides were specific to disrupting the interactions between DNMT1 and DMAP1, DNMT3b, PCNA, EZH2, HDAC1, Sp1 and HP1β, respectively. For example, we observed in Astro#40 that the 197–212 peptide disrupted the DNMT1/DNMT3B interaction, but not the DNMT1/HP1β, DNMT1/HDAC1, DNMT1/Sp1, DNMT1/EZH2 and DNMT1/DMAP1 interactions (Figure 1B). More generally, we noted that peptides designed to specifically disrupt an interaction disrupted only the targeted and expected interactions in Astro#40 and U87 cells ( 2: Figure S2).

### Impact of the disruption of DNMT1/protein-x interactions on the global DNA methylation level

We then investigated the impact of these disruptions on the global level of DNA methylation, i.e., on the 5-me-thylcytosine level. Two DNMT inhibitors (5aza-2deoxy-cytidine and procainamide) were used as these drugs induced global DNA hypomethylation (Figure 1C). In agreement with our previous reports, the 163–174 plasmid was used as a peptide that induced global DNA hypomethylation [3,4] (Figure 1C). Thus, we noted that the disruption of DNMT1/HDAC1 and DNMT1/HP1β interactions promoted a global decrease in the 5-me-thylcytosine level in Astro#40 and U87 cells, while the disruption of the DNMT1/EZH2, DNMT1/Sp1 and DNMT1/DMAP1 interactions did not affect the 5-me-thylcytosine level in these cells (Figure 1C).

### Impact on tumorigenesis of the disruption of DNMT1/protein-x interactions

**Figure 2:**
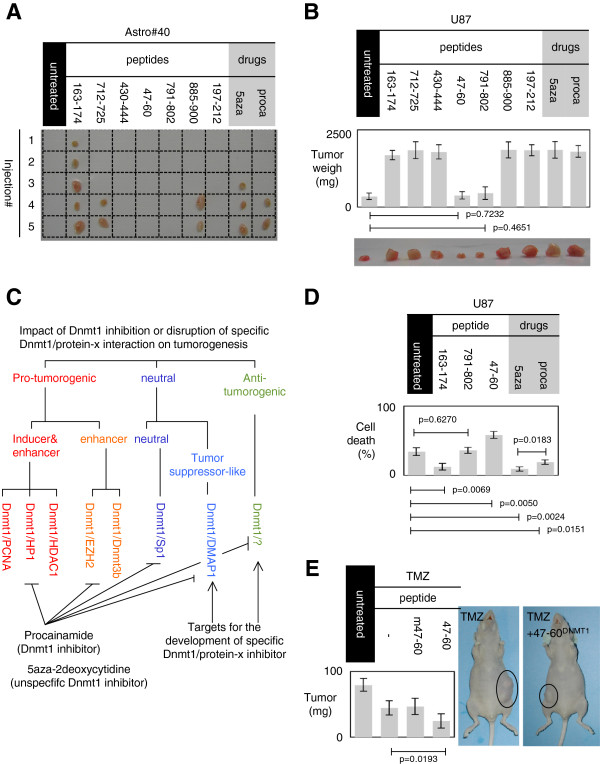
**Impact of specific inhibition of DNMT1/protein-x interaction.** (**A**) Tumorigenicity test of Astro#40 cells transfected with plasmids encoding for indicated peptides or pre-treated with procainamide. For each condition, five subcutaneous injections of 10^6^ cells were performed in Swiss nude nice. Picture illustrates the tumor obtained (or not) 5 weeks after injections. (**B**) Tumorigenicity test of U87 cells transfected with plasmids encoding for indicated peptides or pre-treated with epigenetic drugs. For each condition, three subcutaneous injections of 10^6^ cells were performed in Swiss nude mice. *Graph* illustrates the average ± SD of these data. Picture illustrates representative tumors obtained 4 weeks after injections. (**C**) Effect of different DNMT inhibitor strategies. DNA methylation governs the expression of tumor suppressor genes (TSG) but also of oncogenes and DNA repeat element and retrotransposons (whose demethylation and/or expression promotes the tumorigenesis). By using an unspecific DNMT inhibitors (5aza-2deoxycytidine) or a specific DNMT1 inhibitor (procainamide), we postulate that we induced the hypomethylation not only of TSG but also of the oncogenes and retrotransposons. By reducing the effect of DNMT1 inhibition to certain specific DNMT/protein-x interactions, we still keep the opportunity to promote the expression of the TSG without promoting the hypomethylation-induced activation of retrotransposons and oncogenes. (**D**) Analysis of TMZ + irradiation-induced cell death in U87 cells. Percentages of cell death were evaluated by using a trypan blue stain 0.4%, and the Countess® Automated Cell Counter (Life Technology, France). Graph illustrates the average ± SD of three independent experiments. (**E**) Impact of the addition of 47-60^DNMT1^ on TMZ treatment in an *in vivo* model of glioma. After the tumor establishment, mice were treated with TMZ and TMZ + 47-60^DNMT1^ (Additional file 4: Figure S4). Two negative controls were used: “untreated” represents a treatment with DMSO “m47-60^DNMT1^” symbolized a co-treatment using TMZ and mutated 47-60^DNMT1^. Graph illustrates the average ± SD of five independent experiments.

Tumorigenic assays performed in Swiss nude mice revealed that the subcutaneous injection of Astro#40 cells treated 4 weeks with 5aza-2deoxycytidine and procainamide or transfected with plasmid encoding for the 163–174, 712–725 and 885–900 peptides generated tumor formation in 3/5, 2/5, 5/5, 2/5 and 2/5 cases, respectively (Figure 2A). Thus, we observed that gliomagenesis is induced by the loss of DNMT1/PCNA, DNMT1/HDAC1 and DNMT1/HP1β interactions, i.e., under conditions that also induced a decrease in the global DNA methylation level. In parallel, we noted that the subcutaneous injections of U87 cells treated with 5aza-2deoxycytidine and procainamide or transfected with plasmid encoding for the 163–174, 712–725, 430–444, 885–900 and 197–212 peptides generated the formation of larger tumors as compared to untreated U87 cells (Figure 2B). These data suggest that the disruption of DNMT1/PCNA, DNMT1/EZH2, DNMT1/DNMT3B, DNMT1/HDAC1 and DNMT1/HP1β interactions acts such as an enhancer of tumorigenesis. Thus, our work stratifies the specific disruption of certain DNMT1/protein-x interactions into two categories (Figure 2C). The first category includes the specific disruption of certain DNMT1/protein-x interactions acting as inducers and/or enhancers of gliomagenesis (DNMT1/PCNA, DNMT1/HDAC1 and DNMT1/HP1β, DNMT1/EZH2, DNMT1/DNMT3b), i.e., acting as pro-tumorigenic-specific disruption of DNMT1/protein-x interactions. The second category includes the specific disruption of DNMT1/protein-x interactions devoid of any action on gliomagenesis. This category is called neutral-tumorigenic-specific disruption of DNMT1/protein-x interactions. Although absent in our data, we added a third category called anti-tumorigenic-specific disruption of DNMT1/protein-x interactions including the specific disruption of DNMT1/protein-x interactions promoting the decrease in tumorigenesis (Figure 2C).

### Focus on two disruptions of DNMT1/protein-x interactions devoid of action on gliomagenesis

In the absence of any observations of anti-tumorigenic-specific disruptions of DNMT1/protein-x interactions, we next analyzed whether the specific disruption of a DNMT1/protein-x interaction devoid of action on glioma-genesis could have an impact on the response to the standard GBM treatment combining temozolomide (TMZ) and irradiation. For this, we measured the percentage of cell death induced by a TMZ + irradiation treatment of U87 cells transfected with the plasmids encoding for the 47–60 and 791–802 peptides (Additional file 3: Figure S3). We noted that the 47–60 peptide, which induced the disruption of the DNMT1/DMAP1 interaction, increased the percentage of TMZ + irradiation-induced cell death, while the 791–802 peptide, which induced the disruption of the DNMT1/Sp1 interaction, had no effect on the percentage of TMZ + irradiation-induced cell death (Figure 2D). In addition, our data also indicated that the 5aza-2deoxycytidine and procainamide treatments and the 163–174 peptide induced a phenotype resistant to TMZ + irradiation cell death since the percentage of TMZ-irradiation-induced cell death significantly decreased under these conditions (Figure 2D). Taken together, these data indicate that, among the considered peptides and DNMT1 inhibitors, only the specific 47-60-induced disruption of the DNMT1/DMAP1 interaction increased the percentage of TMZ + irradiation-induced cell death without promoting or increasing the tumorigenesis. Thus, we conclude that the specific inhibition of the DNMT1/DMAP1 interaction is a neutral-tumorigenic-specific inhibition of DNMT1/protein-x interaction harboring the capacity to sensitize cells to TMZ + irradiation-induced cell death.

### Disruption of DNMT1/DMAP1 interactions enhances the anti-tumor effect of TMZ treatment in mice

To investigate this point, established tumors were treated with TMZ, TMZ + 47-60^DNMT1^ and mutated 47-60^DNMT1^ (m47-60^DNMT1^) (Additional file 4: Figure S4). Mice received 6 weeks of treatment as in treatment of human GBM, and tumor weight was analyzed 2 weeks after the end of the treatment. As illustrated in Figure 2E, we first noted that TMZ treatment reduced the tumor weigh of U87-induced glioma. Second, we observed that the addition of 47-60^DNMT1^ increased the efficiency of TMZ treatment and decreased tumor growth, while m47-60 ^DNMT1^ had no effect on the efficiency of TMZ treatment (since this peptide does not disrupt the DNMT1/DNMAP1 interaction; Additional file 5: Figure S5). Thus, our data identify 47-60^DNMT1^ as an enhancer of TMZ treatment.

## Discussion

To summarize, our data indicate that the specific inhibition of certain DNMT1/protein-x interactions (DNMT1/PCNA interaction, for example) and the use of a specific DNMT1 inhibitor (procainamide) or unspecific DNMT1 inhibitor (5-aza-2deoxycytidine) could be used as a treatment, acting as inducers and/or enhancers of tumorigenesis. Our data indicate that the specific inhibition of the DNMT1/DMAP1 interaction acts as a tumor suppressor-like event since the disruption of the DNMT1/DMAP1 interaction increased TMZ + irradiation-induced cell death without promoting the initiation and progression of tumorigenesis. Consequently, we distinguish between among the neutral-tumorigenic-specific inhibition of DNMT1/protein-x interaction and the tumor suppressor-like neutral-tumorigenic-specific inhibition of DNMT1/protein-x interaction (Figure 2C).

Using this example, our data underline the necessity to consider the interaction partners of DNMT1 and not only the DNMT1 structure or activity to develop a DNMT1 inhibitor. In other terms, our data introduce, for the first time, the notion of protein/protein inhibition into the development of DNMT inhibitors. Indeed, without being innovative in the development of drugs or small molecules for a therapeutic application, this strategy is novel in the conception/research of DNMT inhibitors since the identification of DNMT inhibitors is, to date, based on docking-based virtual screening methods, the screening of natural products, the design and generation of derivatives of DNMT inhibitors already known, or molecular modeling of DNMT inhibitors by using crystal structure studies of DNMTs [15-19]. Thus, like ABT-737 and MI-129, two compounds targeting specific protein-protein interactions (pro-apoptotic/anti-apopto-tic and p53/MDM2 interactions, respectively) have opened a new area in targeted therapy; our data argue that the targeting of certain DNMT1/protein-x interactions opens a new area in the development of targeted epigenetic therapy.

However, despite its promising character, the development of specific inhibitors of DNMT1/protein-x interactions requires the identification of tumor suppressor-like neutral-tumorigenic-specific inhibition of DNMT1/protein-x interaction or anti-tumorigenic-specific disruption of certain DNMT1/protein-x interactions. Thus, studies are ongoing in our laboratory to identify a DNMT1/protein-x-including complex promoting the methylation-induced silencing of the tumor suppressor gene without being implicated in the methylation-induced silencing of oncogenes.

Concerning the use of 5aza-2deoxycytidine and pro-cainamide, we are aware that the tumorigenesis processes associated with the use of these drugs are obtained after a long exposure. However, 4 weeks is not a long period on the scale of the majority of the chemotherapeutic treatments that last several months. Regardless, our data provide “a warning” concerning the use of DNA demethylating agents as an anti-cancer therapy and provide proof reinforcing the necessity of using specific DNMT inhibitors or unspecific DNMT1 inhibitors with an adequate and optimized dose schedule, as already described by several publications [20,21]. Besides, several publications also argue that the use of DNMT inhibitors could promote oncogene activation [22,23]. Thus, Chik and Szyf [22] report that 5aza-2deoxycytidine activated both silenced tumor suppressor genes and pro-metastatic genes by demethylation, raising the concern that it could promote metastasis [22]. Associated with our results, these data support the idea of developing specific DNMT1, particularly developing a specific inhibitor of DNMT1/protein-x interaction.

## Methods

### Proximity ligation in situ assay (P-LISA)

P-LISA is a technology permitting the visualization of stable and transient interactions at endogenous protein levels directly *in situ*[20]. Briefly, two primary antibodies raised in different species recognize the target antigen or antigens of interest. Species-specific secondary antibodies, called PLA probes, each with a unique short DNA strand attached to it, bind to the primary antibodies. When the PLA probes are in close proximity (< 40 nm), the DNA strands can interact through a subsequent addition of two other circle-forming DNA oligonucleotides. After ligating the two added oligonucleotides, creating a circle DNA molecule, they are amplified via rolling circle amplification. After amplification, several-hundredfold replication of the DNA circle has occurred, and labeled complementary oligonucleotide probes highlight the product. The resulting high concentration of fluorescence in each single-molecule amplification product is easily visible as a distinct bright dot when viewed with a fluorescence microscope.

Cells were cultured for 24 h on cover slips. Cells were then fixed with 4% paraformaldehyde in PBS, pH 7.4, for 15 min at room temperature. Permeabilization was performed with PBS containing 0.5% Triton X-100 for 20 min at room temperature. Blocking, staining, hybridization, ligation, amplification and detection steps were realized according to the manufacturer’s instructions (Olink Bioscience). All incubations were performed in a humidity chamber. Amplification and detection steps were performed in a dark room. Fluorescence was visualized by using the Axiovert 200M microscopy system (Zeiss, Le Pecq, France) with the ApoTome module (X63 and numerical aperture 1.4). Preparations were mounted using ProLong® Gold antifade reagent with DAPI (InVitrogen, France). Picture acquisition was realized in structured illumination microscopy [24]. Finally, the images were analyzed using the freeware “BlobFinder” available for download from http://www.cb.uu.se/~amin/BlobFinder. Thus, we obtained the number of signals per nuclei since nuclei can be automatically identified.

### Plasmid construction and transfection

To express peptides in cells, we subcloned in pcDNA3.3 (Life Technology, France) the sequences encoding for the indicated peptides. In addition, the NLS sequence (PKKKRKV) was added to the sequences encoding for the indicated peptides in order to address the peptides in the nucleus. Tranfections were next realized by using 2.10^5^ cells, 5 μg of plasmid and Lipofectamine™ 2000 reagents (Life Technology, France). Selection was realized by adding 500 μg/ml of Geneticin selective antibiotic in complete medium of cell culture for 3 weeks. Next, 1 week was used to amplify the cells. Veracity of transfection was determined by PCR analyses using primers directed against pcDNA3.3 (ACGTTGTCACTGAAGCGG and CCTGATGCTCTTCGTCCA) and by the fact that each plasmid affects the DNMT1/protein-x interaction of interest.

### Measure of the 5-methylcytosine level

DNA was extracted using the QiaAmp DNA mini Kit (Qiagen, France). The quantification of 5-methylcytosine is performed by using the Methylamp Global DNA methylation Quantification kit (Euromedex-Epigenetiek, France).

### Tumorigenicity assay

Cultured cells were harvested by trypsinization, washed and resuspended in saline buffer. Cell suspensions were injected s.c. as 10^6^ cells in 0.2 ml volume in the flank of 7-/8-week-old nude NMRI-nu female mice (Janvier, France).

## Competing interests

The authors declare that they have no financial relationship with the organization that sponsored the research.

## Authors’ contributions

PFC initiated and conceived the study, undertook and interpreted analyses, and drafted the manuscript. FMV provided data interpretation and manuscript review. PFC, MC, RP and AN performed epigenetic laboratory studies. All authors read and approved the final manuscript.

## Supplementary Material

Additional file 1: Figure S1 Representation of the pcDNA3.3 plasmid used to express the indicated peptides in cells. For each peptide, the amino acid position, sequences and corresponding cDNA sequences are indicated in the table. Click here for file

Additional file 2: Figure S2 Graphs illustrate the impact of the expression of the considered peptide on the indicated DNMT1/protein-x interaction of close proximity. Graph illustrates the average ± SD obtained from 100 cells in three independent experiments. *Only the corresponding interaction was significantly decreased (*p* < 0.05, *t*-test) by the considered peptides in comparison with the data obtained from untreated cells. “Untreated” indicates that cells are not transfected by a plasmid encoding for a peptide or are not treated with a DNMT inhibitor. Thus, this condition is used as a control. For each peptide, the specificity of inhibition of disruption of one considered peptide is reinforced by the use of six other peptides. Click here for file

Additional file 3: Figure S3 Schematic representation of the TMZ + irradiation treatment administered to the cells. Click here for file

Additional file 4: Figure S4 Schematic representation of the treatments administered to mice. Click here for file

Additional file 5: Figure S5 Effect of 47-60^DNMT1^ and m47-60^DNMT1^ on the DNMT1/DMAP1 interaction. Graphs illustrate the impact of the considered peptide on the indicated DNMT1/protein-x interaction of close proximity. Graph illustrates the average ± SD obtained from 100 cells in three independent experiments. *Only the corresponding interaction was significantly decreased (*p* < 0.05, *t*-test) by the considered peptides in comparison with the data obtained from untreated cells. Click here for file

## References

[B1] Eden A Gaudet F Waghmare A Jaenisch R Chromosomal instability and tumors promoted by DNA hypomethylation Science 2003 300 5618 455 10.1126/science.1083557 12702868

[B2] Gaudet F Hodgson JG Eden A Jackson-Grusby L Dausman J Induction of tumors in mice by genomic hypomethylation Science 2003 300 5618 489 492 10.1126/science.1083558 12702876

[B3] Hervouet E Lalier L Debien E Cheray M Geairon A Tumor induction by disruption of the Dnmt1, PCNA and UHRF1 interactions Nature Precedings 2008 http://hdl.handle.net/10101/npre.2008.2509.1

[B4] Hervouet E Debien E Cheray M Hulin P Loussouarn D Disruption of Dnmt1/PCNA/UHRF1 interactions promotes tumorigenesis by inducing genome and gene-specific hypomethylations and chromosomal instability PLoS One 2010 5 6 e11333 10.1371/journal.pone.0011333 20613874 PMC2894052

[B5] Bostick M Kim J Estève P Clark A Pradhan S UHRF1 plays a role in maintaining DNA methylation in mammalian cells Science 2007 27 15 2187 2197 10.1126/science.1147939 17673620

[B6] Estève P Chin H Pradhan S Human maintenance DNA (cytosine-5)-methyltransferase and p53 modulate expression of p53-repressed promoters Proc Natl Acad Sci U S A 2005 102 4 1000 1005 10.1073/pnas.0407729102 15657147 PMC544618

[B7] Estève P Chin H Pradhan S Molecular mechanisms of transactivation and doxorubicin-mediated repression of survivin gene in cancer cells J Biol Chem 2007 282 4 2616 2625 10.1074/jbc.M606203200 17124180

[B8] Fuks F Hurd P Deplus R Kouzarides T The DNA methyltransferases associate with HP1 and the SUV39H1 histone methyltransferase Nucleic Acids Res 2003 31 9 2305 2312 10.1093/nar/gkg332 12711675 PMC154218

[B9] Fuks F Burgers WA Brehm A Hughes-Davies L Kouzarides T DNA methyltransferase Dnmt1 associates with histone deacetylase activity Nat Genet 2000 24 1 88 91 10.1038/71750 10615135

[B10] Hervouet E Vallette FM Cartron PF Dnmt1/transcription factor interactions: an alternative mechanism of DNA methylation inheritance Genes & Cancer 2010 1 5 434 443 10.1177/1947601910373794 21779454 PMC3092212

[B11] Margot JB Ehrenhofer-Murray AE Leonhardt H Interactions within the mammalian DNA methyltransferase family BMC Mol Biol 2003 4 1 7 10.1186/1471-2199-4-7 12777184 PMC166133

[B12] Muromoto R Sugiyama K Takachi A Imoto S Sato N Physical and functional interactions between Daxx and DNA methyltransferase 1-associated protein, DMAP1 J Immunol 2004 172 5 2985 2993 14978102 10.4049/jimmunol.172.5.2985

[B13] Sharif J Muto M Takebayashi S Suetake I Iwamatsu A The SRA protein Np95 mediates epigenetic inheritance by recruiting Dnmt1 to methylated DNA Nature 2007 450 7171 908 912 10.1038/nature06397 17994007

[B14] Viré E Brenner C Deplus R Blanchon L Fraga M The Polycomb group protein EZH2 directly controls DNA methylation Nature 2006 439 7078 871 874 16357870 10.1038/nature04431

[B15] Kuck D Singh N Lyko F Medina-Franco J Novel and selective DNA methyltransferase inhibitors: docking-based virtual screening and experimental evaluation Bioorg Med Chem 2010 18 2 822 829 10.1016/j.bmc.2009.11.050 20006515

[B16] Medina-Franco J López-Vallejo F Kuck D Lyko F Natural products as DNA methyltransferase inhibitors: a computer-aided discovery approach Mol Divers 2011 15 2 293 304 10.1007/s11030-010-9262-5 20697809

[B17] Suzuki T Tanaka R Hamada S Nakagawa H Miyata N Design, synthesis, inhibitory activity, and binding mode study of novel DNA methyltransferase 1 inhibitors Bioorg Med Chem Lett 2010 20 3 1124 1127 10.1016/j.bmcl.2009.12.016 20056538

[B18] Yoo J Medina-Franco J Inhibitors of DNA methyltransferases: insights from computational studies Curr Med Chem 2012 19 21 3475 3487 10.2174/092986712801323289 22709005

[B19] Yoo J Kim J Robertson K Medina-Franco J Molecular modeling of inhibitors of human DNA methyltransferase with a crystal structure: discovery of a novel DNMT1 inhibitor Adv Protein Chem Struct Biol 2012 87 219 247 22607757 10.1016/B978-0-12-398312-1.00008-1 PMC3837394

[B20] Issa J Optimizing therapy with methylation inhibitors in myelodysplastic syndromes: dose, duration, and patient selection Nat Clin Pract Oncol 2005 2 1 S24 S29 16341237 10.1038/ncponc0355

[B21] Momparler R Côté S Eliopoulos N Pharmacological approach for optimization of the dose schedule of 5-Aza-2'-deoxycytidine (Decitabine) for the therapy of leukemia Leukemia Suppl 2007 1 S1 S6 9130684

[B22] Chik F Szyf M Effects of specific DNMT gene depletion on cancer cell transformation and breast cancer cell invasion; toward selective DNMT inhibitors Carcinogenesis 2010 32 2 224 232 20980350 10.1093/carcin/bgq221

[B23] Liu L Ling X Liang H Gao Y Yang H Hypomethylation mediated by decreased DNMTs involves in the activation of proto-oncogene MPL in TK6 cells treated with hydroquinone Toxicol Lett 2012 209 3 239 245 10.1016/j.toxlet.2011.12.020 22245671

[B24] Schaefer L Schuster D Schaffer J Structured illumination microscopy: artefact analysis and reduction utilizing a parameter optimization approach J Microsc 2004 216 Pt 2 165 174 15516228 10.1111/j.0022-2720.2004.01411.x

