# Supplementary material for: Specific inhibition of one DNMT1-including complex influences tumor initiation and progression
Source: Clin Epigenetics. 2013 Jun 28;5(1):9. doi: 10.1186/1868-7083-5-9 (PMC3727981; doi:10.1186/1868-7083-5-9)
Supplement: Additional file 1: Figure S1 — Representation of the pcDNA3.3 plasmid used to express the indicated peptides in cells. For each peptide, the amino acid position, sequences and corresponding cDNA sequences are indicated in the table. [file 1868-7083-5-9-S1.pdf]

	Animo acids position in Dnmt1	Amino acid sequence	cDNA sequence	interactions
Peptide	47-60	NLLHEFLQTEIKNQ	AATTTATTACATGAATTTTTACAAAC CGAAATTAAAAATCAA	Dnmt1/DMAP1
	197-212	DKDQDEKRRRVTSRER	GATAAAGATCAAGATGAAAAACGCC GCCGCGTTACTTCCCGCGAACGC	Dnmt1/Dnmt3b
	163-174	RQTTITSHFAKG	CGCCAAACTACTATTACTTCCCATTT TGCTAAAGGT	Dnmt1/PCNA
	430-444	NIELFFSGSAKPIYD	AATATTGAATTATTTTTTTCCGGTTC CGCTAAACCTATTTATGAT	Dnmt1/EZH2
	712-725	MPSPKKMHQGKKKK	ATGCCAAGTCCAAAGAAGATGCACC AGGGAAAGAAGAAGAAG	Dnmt1/HDAC1
	791-802	MFHAHWFCAGTD	ATGTTTCATGCTCATTGGTTTTGTGC TGGTACTGAT	Dnmt1/Sp1
	885-900	TEDNKFKFCVSCARLA	ACAGAGGACAACAAGTTCAAGTTCT GCGTAAGTTGCGCAAGACTAGCA	Dnmt1/HP1β

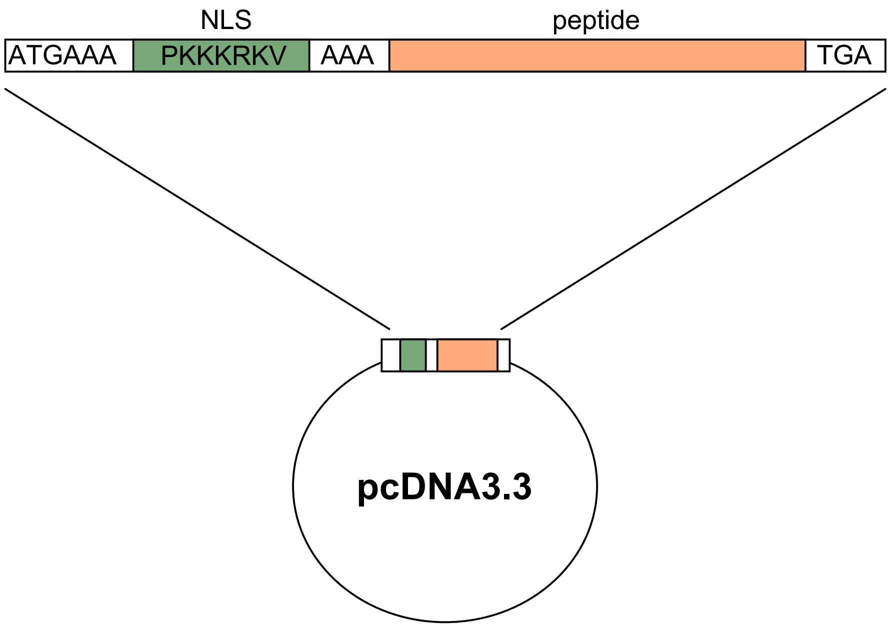

Figure S1
