# Supplementary figures and images for: Specific inhibition of one DNMT1-including complex influences tumor initiation and progression
Source: Clin Epigenetics. 2013 Jun 28;5(1):9. doi: 10.1186/1868-7083-5-9 (PMC3727981; doi:10.1186/1868-7083-5-9)

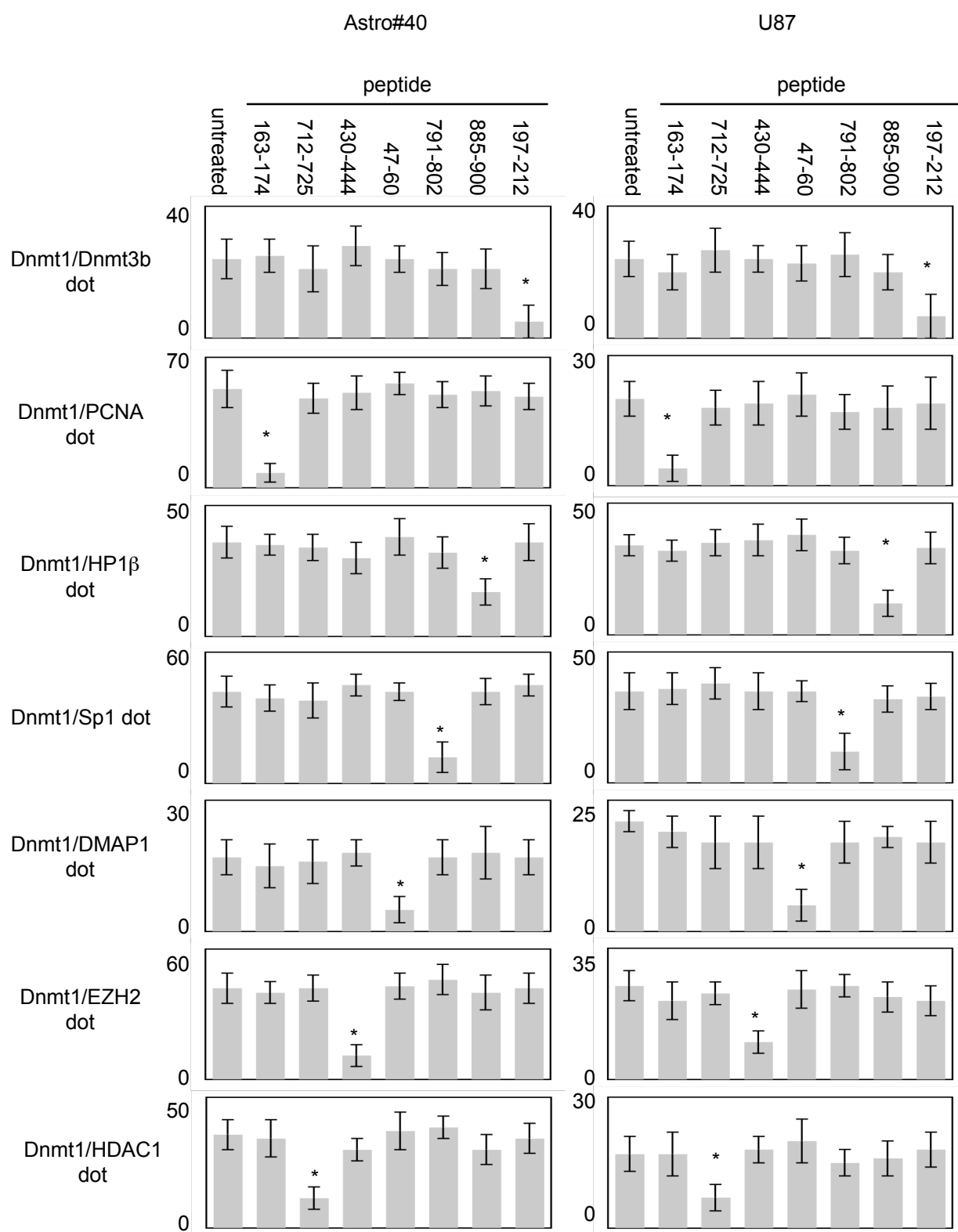

**Figure S2**

Supplement: Additional file 2: Figure S2 — Graphs illustrate the impact of the expression of the considered peptide on the indicated DNMT1/protein-x interaction of close proximity. Graph illustrates the average ± SD obtained from 100 cells in three independent experiments. *Only the corresponding interaction was significantly decreased (p < 0.05, t-test) by the considered peptides in comparison with the data obtained from untreated cells. “Untreated” indicates that cells are not transfected by a plasmid encoding for a peptide or are not treated with a DNMT inhibitor. Thus, this condition is used as a control. For each peptide, the specificity of inhibition of disruption of one considered peptide is reinforced by the use of six other peptides. [file 1868-7083-5-9-S2.pdf]

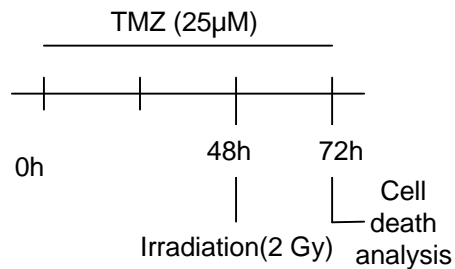

**Figure S3**

Supplement: Additional file 3: Figure S3 — Schematic representation of the TMZ + irradiation treatment administered to the cells. [file 1868-7083-5-9-S3.pdf]

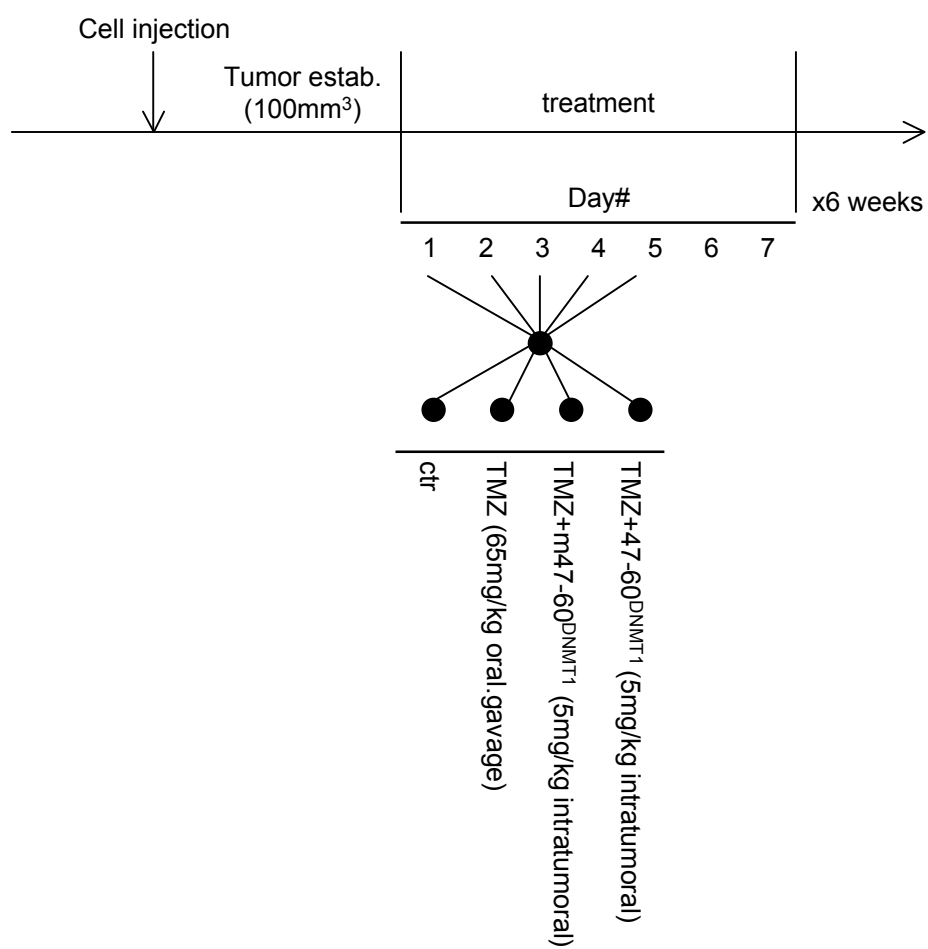

**Figure S4**

Supplement: Additional file 4: Figure S4 — Schematic representation of the treatments administered to mice. [file 1868-7083-5-9-S4.pdf]

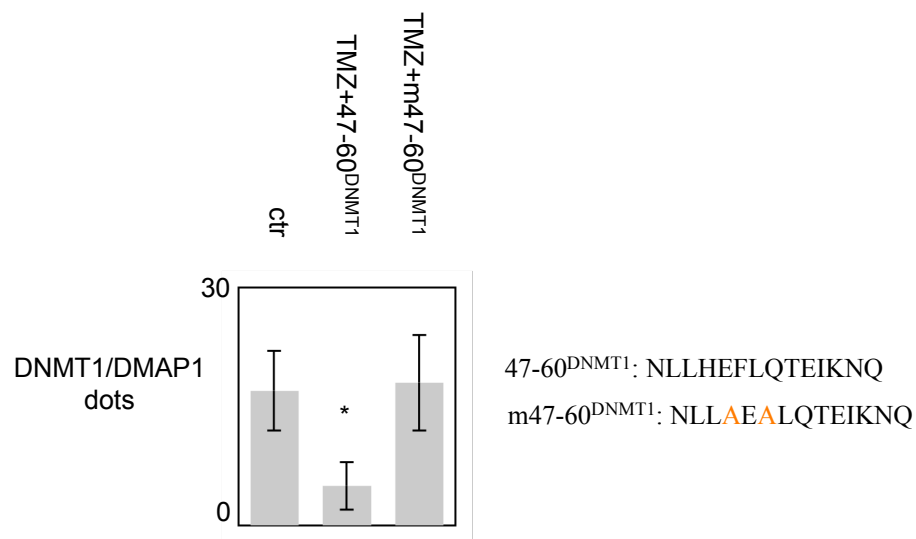

**Figure S5**

Supplement: Additional file 5: Figure S5 — Effect of 47-60DNMT1 and m47-60DNMT1 on the DNMT1/DMAP1 interaction. Graphs illustrate the impact of the considered peptide on the indicated DNMT1/protein-x interaction of close proximity. Graph illustrates the average ± SD obtained from 100 cells in three independent experiments. *Only the corresponding interaction was significantly decreased (p < 0.05, t-test) by the considered peptides in comparison with the data obtained from untreated cells. [file 1868-7083-5-9-S5.pdf]
